# Subcutaneous fibroblastoma resembling hemangioma: A case of benign tumor of the chest wall

**DOI:** 10.1002/ccr3.7523

**Published:** 2024-03-31

**Authors:** Rabbia Zubair, Muhammad Hassan Zulfi, Úzair Yaqoob, Ka Yiu Lee

**Affiliations:** ^1^ Department of Surgery Hamdard University Hospital Karachi Pakistan; ^2^ Department of Orthopedic Surgery Jinnah Postgraduate Medical Center Karachi Pakistan; ^3^ Department of Health Sciences Mid Sweden University Ostersund Sweden

**Keywords:** chest wall tumors, desmoplastic fibroblastoma, hemangioma, soft tissue tumors, subcutaneous fibroblastoma

## Abstract

**Key Clinical Message:**

Desmoplastic fibroma presents similar to other soft tissue tumors to such an extent that even a gold standard investigation can miss.

**Abstract:**

This is to report a mass in a 47‐year‐old male arising from the chest wall, which was first thought to be a hemangioma but was later diagnosed as a case of desmoplastic fibroblastoma with the help of a biopsy.

## Graphical abstract

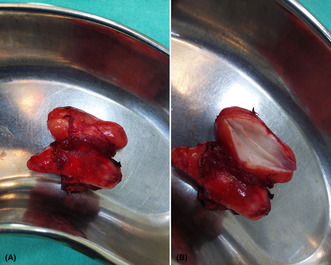

## 1 INTRODUCTION

Desmoplastic fibroblastoma (DF) is a rare soft tissue benign neoplasm which was first described by Evans in 1995 and was named “collagenous fibroma (CF)” in the following year by Nielsen et al.^1^, ^2^ According to the 2020 World Health Organization classification of soft tissue tumors, DF falls under the fibroblastic/myofibroblastic tumor group category.^1^ Some authors consider DF as “semi‐malignant” because it can sometimes behave as a locally aggressive tumor. For this reason, it was cataloged as a “distinct entity” by Jaffe for the first time.^3^

DF holds a high incidence rate in the fifth to seventh decades of life, predominantly affecting males.^1^ Anatomically, lesions have a wide range of distribution, but it occurs mostly in the shoulder girdle, posterior neck, upper back, abdominal wall, limbs, parotid gland, and rarely in the tongue and palate.^4^, ^5^ It presents as a painless subcutaneous mass that is slow‐growing, mobile, and firm, of longer duration, and is commonly involving the skeletal and facial muscles.^1^

DF is a paucicellular, hypovascular tumor characterized by the presence of bland spindle or stellate‐shaped fibroblastic cells that are meagerly distributed in a collagenous/fibroedematous stroma.^6^, ^7^ DF lesions show minimal or absent mitotic activity with no necrosis.^5^ Occasionally, DF exhibits invasive characteristics despite being a benign neoplasm. However, surgical excision remains the therapy of choice as no recurrence has been documented, even for invasive DF.^1^

We report a rare case of DF in a 47‐year‐old male arising from the chest wall, which was first thought to be a hemangioma at the time of presentation but was later diagnosed as a case of DF with the help of a biopsy.

## 2 CASE PRESENTATION

A 47‐year‐old male with no known comorbids came through the outpatient department with a complaint of swelling at the left side of his upper chest since birth and pain at the swelling for 20–25 days.

The swelling increased in size in the last 20 days. The pain was sudden in onset, mild to moderate, dull, radiating to the left shoulder, and aggravated by hot weather.

He was a Naswar addict for 10–12 years. The rest of the history was nonsignificant.

On examination, there was a swelling on the left sternoclavicular region. The covering skin was normal with no color changes or discharge. The temperature of the skin was also normal. The swelling was firm in consistency, with no fluctuation or compressibility, lobulated, non‐tender, immobile, and adherent to the underlying structures but not to the skin.

**FIGURE 1:**
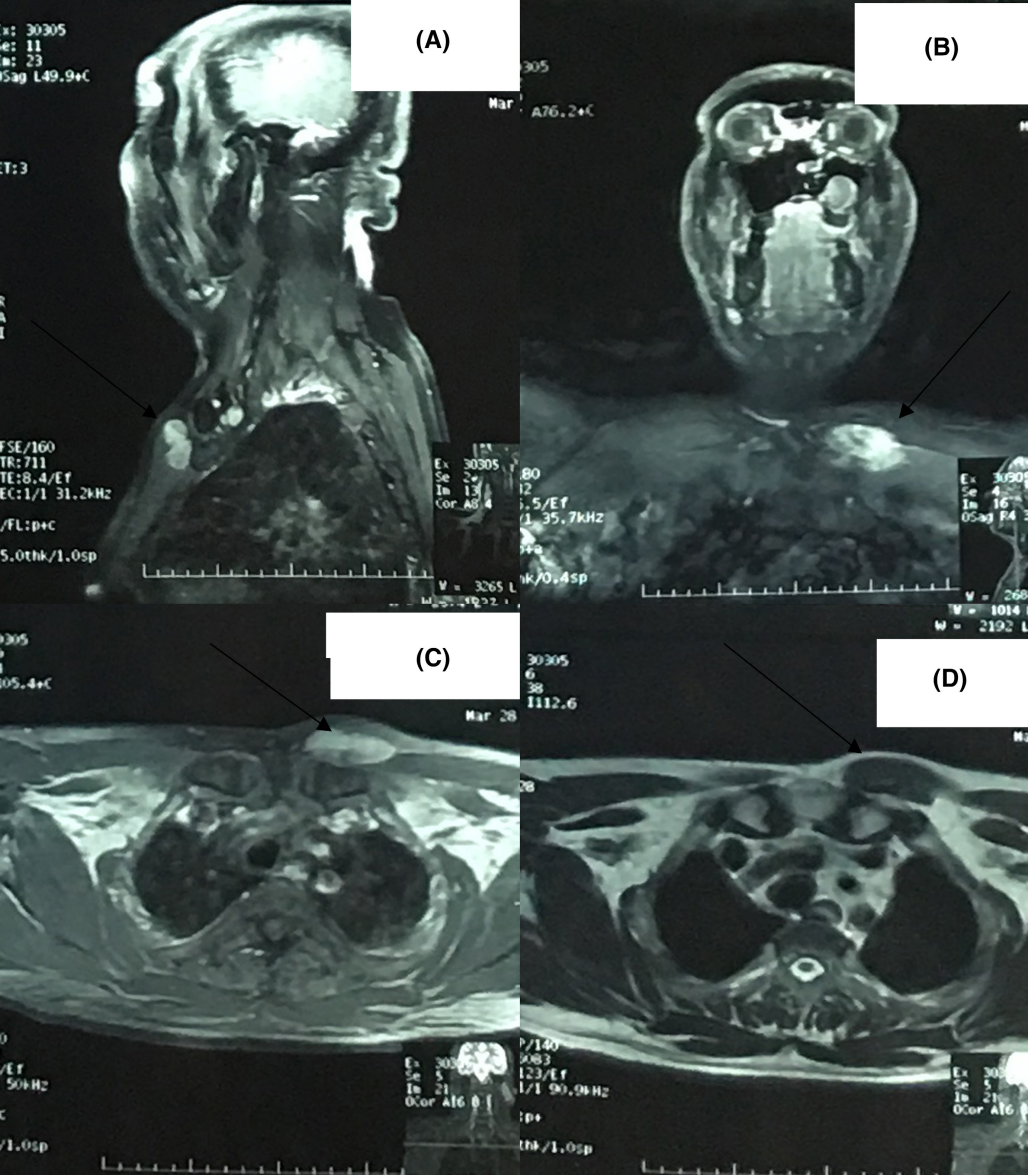
Magnetic resonance image of the chest and neck showing an abnormal signal intensity lesion identified subcutaneously at the level of medial end of clavicle causing a focal bulge anteriorly, abutting the left pectoralis major with no bony changes. (A) Sagittal sequence; (B) Coronal sequence showing neck; (C) Axial T1W imaging; (D) Axial T2W imaging.

MRI of the swelling was done which showed evidence of abnormal signal intensity subcutaneous lesion identified at the level of the medial end of the clavicle measuring 5.3 × 2.5 × 1.9 cm (TR × AP × CC). It was causing a focal bulge anteriorly closely abutting the left pectoralis major. There were no bony changes in the left clavicle and no fat stranding was seen in the adjacent subcutaneous fat. The lesion showed homogenous enhancement on post‐contrast imaging suggestive of a benign etiology, possibly soft tissue hemangioma (Figure 1). Visualized cervical spine shows T2/T1W hyperintense area within the C7 and D4 vertebral bodies likely hemangiomas. Multiple posterior disc osteophyte complexes were noted in the cervical spine from C3‐C4 to C6‐C7 level causing significant spinal canal and bilateral neural foraminal narrowing with radicular compression noted at these levels.

Under informed consent, the patient was prepared for excision under general anesthesia.

General anesthesia was given. After maintaining all aseptic measures and proper draping of the patient, an elliptical incision was made around the swelling. Skin, subcutaneous tissue, and pectoralis major muscle incised. The swelling was identified and excised in toto along with the surrounding muscle. Hemostasis was secured and the wound closed in layers. An aseptic dressing was done.

**FIGURE 2:**
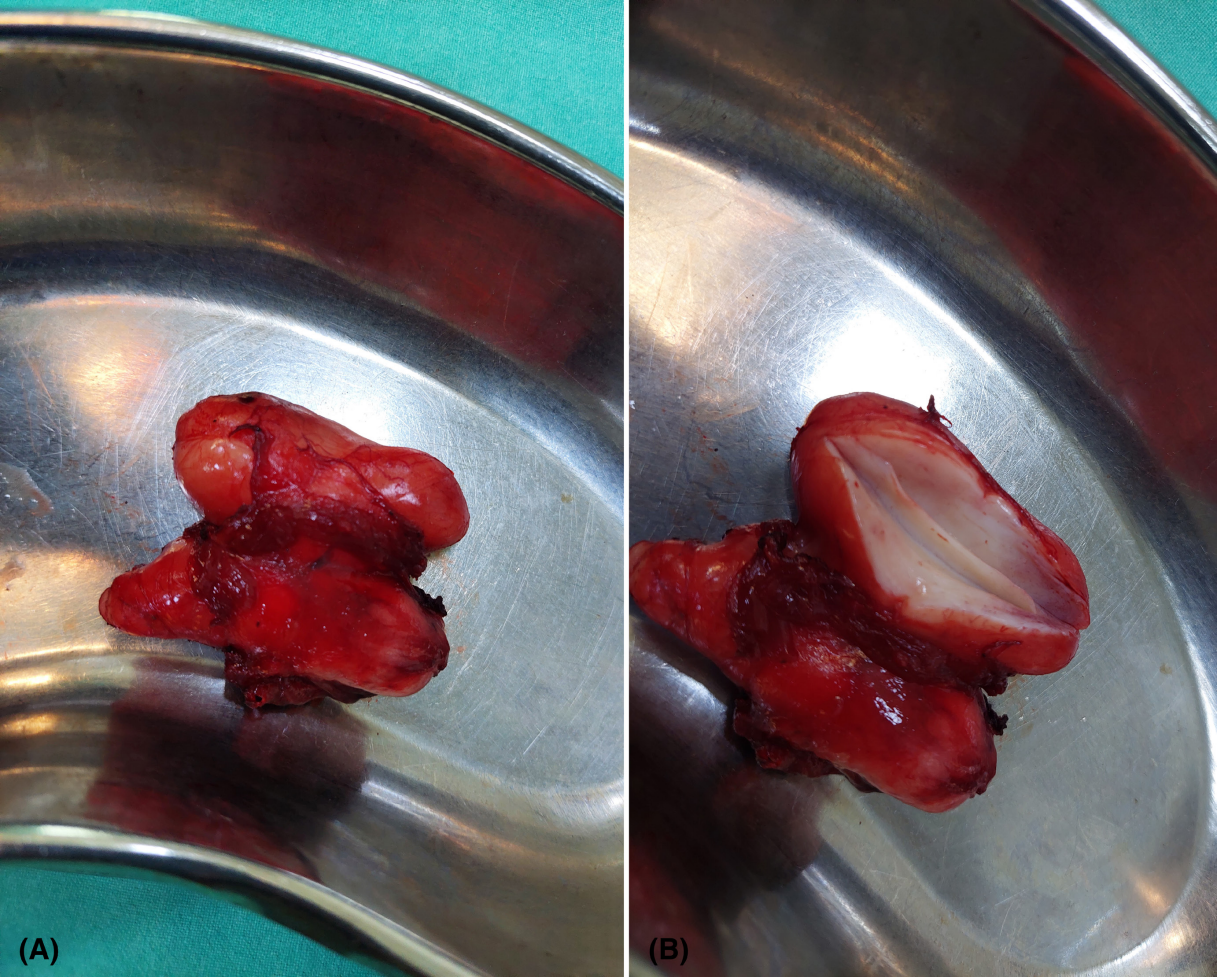
(A, B) Excised mass along with some surrounding muscle and a cut section (1B).

Operative findings were an approximately 4 × 4 cm, firm to hard, lobulated swelling buried inside the pectoralis major muscle (Figure 2).

**FIGURE 3:**
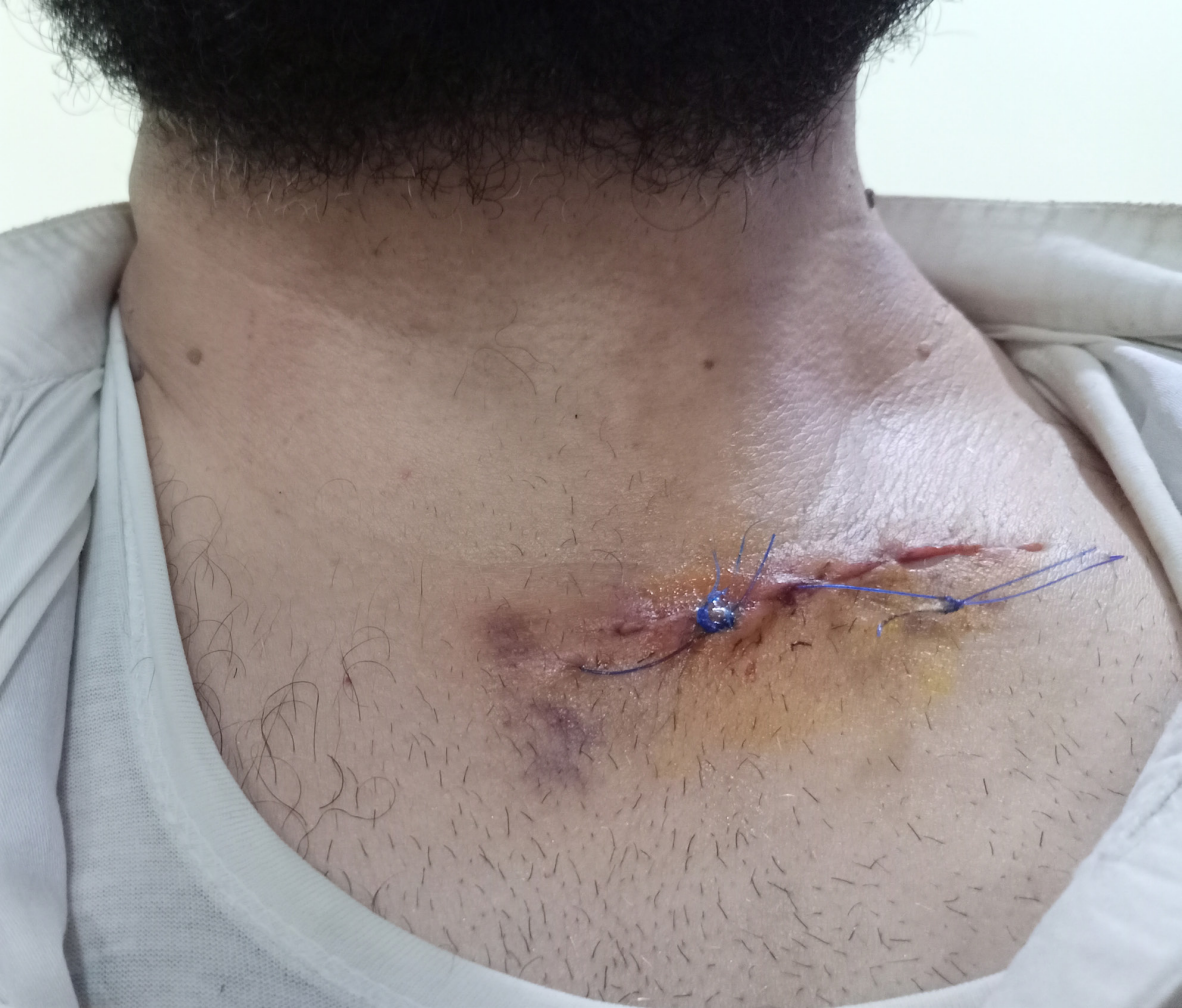
Patient's surgical site presentation during a follow‐up visit.

The patient remained stable postoperatively with no complications. He was discharged on oral amoxiclav tablet for 3 days along with analgesics to be followed up weekly for a month then monthly for 6 months. All follow‐up visits went uneventful with no complaints. The wound healed normally with a little swelling visible for a week but with no infection, hematoma, or any other complications (Figure 3).

Excised mass was sent for histopathology which on gross examination revealed a multilobar lump measuring 5.5 × 4.5 × 2 cm. The margins were tumor‐free with a minimum distance of 0.6 cm. The cut surface of the nodule was gray‐white and firm in consistency. Microscopic examination showed a circumscribed hypocellular spindle‐shaped lesion with heavily collagenized stroma. The cells had stellate nuclei with bland features and no mitoses. There were scattered thin‐walled vessels in the stroma. No infiltration was seen in the peripheral areas. There was no necrosis. The conclusion after consulting a senior pathologist was that this was a benign spindle‐shaped tumor, favoring desmoplastic fibroma/fibroblastoma, negative for malignancy.

## 3 DISCUSSION

DF is a rare, benign, primary, slow‐growing, locally aggressive soft tissue neoplasm.^1^, ^8^, ^9^ It has been reported to have a peak incidence rate in the fifth to seventh decades of life, predominantly in males.^1^ To the best of our knowledge, only one case of DF evolving from the chest wall has been documented to date, however, it can affect any bone in the body.^8^, ^9^ The preoperative diagnosis is difficult and may be confused with other benign or malignant spindle cell neoplasms of the soft tissue.^6^, ^9^ The radiological features of other bone lesions like hemangioma, low‐grade osteosarcoma, eosinophilic granuloma, and fibrous dysplasia are similar to those of DF.^8^ DF also has a similar presentation to that of hamartoma, therefore, proper investigations are required for attaining a definitive diagnosis.^10^

In our case, the patient presented with a complaint of swelling, which was misdiagnosed as a hemangioma. An MRI of the swelling was done, which revealed a subcutaneous lesion with abnormal signal intensity at the level of the medial end of the clavicle. There were no bony changes in the left clavicle, and the nearby subcutaneous fat exhibited no evidence of fat trapping. On post‐contrast imaging, the lesion showed homogenous enhancement, suggesting a benign etiology, possibly a soft tissue hemangioma. Visualized cervical spine showed a T2/T1W hyperintense area within the C7 and D4 vertebral bodies, likely hemangiomas, which were in congruence with the MR findings of studies done by Sakurai et al. and Gaudino et al.^11^, ^12^

The mass was then excised and sent for biopsy. Although hamartoma can present like DF, its microscopic features which consist of adipocytes and isolated chondrocytes surrounded by a rich matrix emerging within the lacunar spaces, as described in the study by Ali et al., were not in congruence with those mentioned in our report, and it was therefore ruled out of our differentials.^10^ The histopathological findings observed in our patient were similar to those described by Watanabe et al., Okubo et al., and Kato et al.^6^, ^7^, ^8^ The cut surface of the nodule was gray‐white and firm in consistency. On microscopic analysis, a hypocellular spindle‐shaped lesion with a defined border and extensively collagenized stroma was seen. The cells lacked mitoses and possessed stellate nuclei with bland features. There were scattered thin‐walled vessels in the stroma. No necrosis or infiltration was seen in the surrounding areas. These findings were confirmatory for the diagnosis of DF.

Most studies have documented that MR imaging contributes insignificantly to the differential diagnosis. As the signal intensities of the tumor are said to be non‐specific, MR imaging is considered ineffective in characterizing DF.^3^ This can explain why MRI was unable to differentiate DF from hemangioma in our case.

Curettage or intralesional excision results in a local recurrence rate of at least 40%, therefore, total excision is the treatment of choice for DF.^8^, ^9^, ^13^ Our patient had a successful resection of the tumor and showed no signs of recurrence 6 months following surgery.

## 4 CONCLUSION

We conclude that DF, although rare, shares common features with other soft tissue tumors like hemangiomas to such an extent that even a gold standard investigation like MR imaging was unable to identify it as a case of DF until the mass was excised and sent for histopathological evaluation. As far as the management of this benign neoplasm is concerned, total surgical excision remains the therapy of choice, with no recurrence reported in postoperative follow‐up.

## AUTHOR CONTRIBUTIONS

**Rabbia Zubair:** Conceptualization; data curation; formal analysis; funding acquisition; investigation; methodology; project administration; resources; software; supervision; validation; visualization; writing – original draft; writing – review and editing. **Muhammad Hassan Zulfi:** Conceptualization; data curation; formal analysis; funding acquisition; investigation; methodology; project administration; resources; software; supervision; validation; visualization; writing – original draft; writing – review and editing. **Uzair Yaqoob:** Conceptualization; data curation; formal analysis; funding acquisition; investigation; methodology; project administration; resources; software; supervision; validation; visualization; writing – original draft; writing – review and editing. **Ka Yiu Lee:** Visualization; writing – review and editing.

## FUNDING INFORMATION

The Corresponding Author's institution or funder has an existing agreement (pre‐paid open access account) with Wiley and may pay the article publication charge from this account on behalf of the author, or offer a discounted APC.

## CONFLICT OF INTERESTS STATEMENT

The authors declare that there is no conflict of interest.

## CONSENT

Written informed consent was obtained from the patient to publish this report in accordance with the journal's patient consent policy.

## Data Availability

Data sharing is not applicable to this article as no new data were created or analyzed in this study.
